# Acute SARS-CoV-2 Infection is Highly Cytopathic, Elicits a Robust Innate Immune Response and is Efficiently Prevented by EIDD-2801

**DOI:** 10.21203/rs.3.rs-80404/v1

**Published:** 2020-09-24

**Authors:** Angela Wahl, Lisa Gralinski, Claire Johnson, Wenbo Yao, Martina Kovarova, Kenneth Dinnon, Hongwei Liu, Victoria Madden, Halina Krzystek, Chandrav De, Kristen White, Alexandra Schäfer, Tanzila Zaman, Sarah Leist, Paul Grant, Kendra Gully, Frederic Askin, Edward Browne, Corbin Jones, Raymond Pickles, Ralph Baric, J Victor Garcia

**Affiliations:** International Center for the Advancement of Translational Science, Division of Infectious Diseases, Department of Medicine, and Center for AIDS Research, University of North Carolina at Chapel Hill; UNC; University of North Carolina-Chapel Hill; University of North Carolina-Chapel Hill; University of North Carolina-Chapel Hill; University of North Carolina at Chapel Hill; University of North Carolina-Chapel Hill; University of North Carolina; University of North Carolina-Chapel Hill; International Center for the Advancement of Translational Science, Division of Infectious Diseases, Department of Medicine, and Center for AIDS Research, University of North Carolina at Chapel Hill; University of North Carolina-Chapel Hill; University of North Carolina at Chapel Hill; University of North Carolina-Chapel Hill; University of North Carolina at Chapel Hill; University of North Carolina-Chapel Hill; University of North Carolina at Chapel Hill; University of North Carolina-Chapel Hill; UNC HIV Cure Center, Division of Infectious Diseases, Department of Medicine, and Center for AIDS Research, University of North Carolina; University of North Carolina at Chapel Hill; University of North Carolina-Chapel Hill; University of North Carolina; University of North Carolina at Chapel Hill

**Keywords:** COVID-19, SARS-CoV-2, virus replication, endogenous bat coronaviruses

## Abstract

All known recently emerged human coronaviruses likely originated in bats. Here, we used a single experimental platform based on human lung-only mice (LoM) to demonstrate efficient in vivo replication of all recently emerged human coronaviruses (SARS-CoV, MERS-CoV, SARS-CoV-2) and two highly relevant endogenous pre-pandemic SARS-like bat coronaviruses. Virus replication in this model occurs in bona fide human lung tissue and does not require any type of adaptation of the virus or the host. Our results indicate that bats harbor endogenous coronaviruses capable of direct transmission into humans. Further detailed analysis of pandemic SARS-CoV-2 in vivo infection of LoM human lung tissue showed predominant infection of human lung epithelial cells, including type II pneumocytes present in alveoli and ciliated airway cells. Acute SARS-CoV-2 infection was highly cytopathic and induced a robust and sustained Type I interferon and inflammatory cytokine/chemokine response. Finally, we evaluated a pre-exposure prophylaxis strategy for coronavirus infection. Our results show that prophylactic administration of EIDD-2801, an oral broad spectrum antiviral currently in phase II clinical trials for the treatment of COVID-19, dramatically prevented SARS-CoV-2 infection in vivo and thus has significant potential for the prevention and treatment of COVID-19.

## Main Text

The recently emerged human pandemic coronavirus Severe Acute Respiratory Syndrome coronavirus-2 (SARS-CoV-2), the causative agent of coronavirus disease (COVID)-19, has spread to six continents resulting in substantial morbidity and mortality worldwide^1^. Bats serve as a natural reservoir for coronaviruses and are the presumed source of SARS-CoV-2 and the highly pathogenic human coronaviruses SARS-CoV and Middle East Respiratory Syndrome (MERS)-CoV^2^. Transmission of coronaviruses from bats to other species is well-documented and adaptation in an intermediary host can facilitate their transmission to humans^2^. While it is possible that SARS-CoV-2 was transmitted to humans via an intermediate host such as pangolins, phylogenic analysis indicates that the SARS-CoV-2 lineage has circulated in bats for decades and evolved in bats into a virus capable of replicating in human cells^3^. Thus, bats are a potential reservoir for coronaviruses with human pandemic potential that can be directly transmitted to humans. Given the repeated and accelerating emergence of highly pathogenic coronaviruses, it has become increasingly important to monitor and characterize coronaviruses circulating in bats and to identify the viral determinants of human infection, disease, and global spread as well as to develop effective therapeutic interventions. Animal models are useful in studying highly pathogenic human coronaviruses, the emergence potential of zoonotic coronaviruses, and to evaluate novel inhibitors for their ability to control coronavirus infection^4–15^. However, human coronaviruses do not replicate in mice without either extensive adaptation of the virus or genetic modification of the host by genetic editing of the receptor or by introducing the individual human receptor genes for each virus^4–11,14,15^. Although existing rodent models have made several important contributions to our understanding of coronavirus infection and pathogenesis, none of these models possess the diverse set of primary human cells present in the human lung that can serve as targets for viral infection^16^. Here, we show that human lung-only mice (LoM), immune deficient mice implanted with authentic human lung tissue^17^, allow for the *in vivo* study of all recently emerged human coronaviruses (SARS-CoV, MERS-CoV, SARS-CoV-2) in a single platform that permits direct comparison of experimental outcomes. Using LoM, we also established that bats harbor novel coronaviruses capable of efficient replication in human lungs without prior adaptation. In addition, we performed an in-depth *in vivo* analysis of acute SARS-CoV-2 infection in the human lung. Our results revealed robust SARS-CoV-2 replication, pathogenesis and sustained activation of the innate host immune response. Finally, we used this platform for the *in vivo* evaluation of EIDD-2801, an orally administered broad spectrum antiviral currently in phase II clinical trials for COVID-19 treatment, to prevent SARS-CoV-2 infection. Our results show that EIDD-2801 efficiently prevented SARS-CoV-2 infection *in vivo* strongly supporting its progression in clinical development for COVID-19.

## Human and bat coronavirus replication in LoM

**Figure 1:**
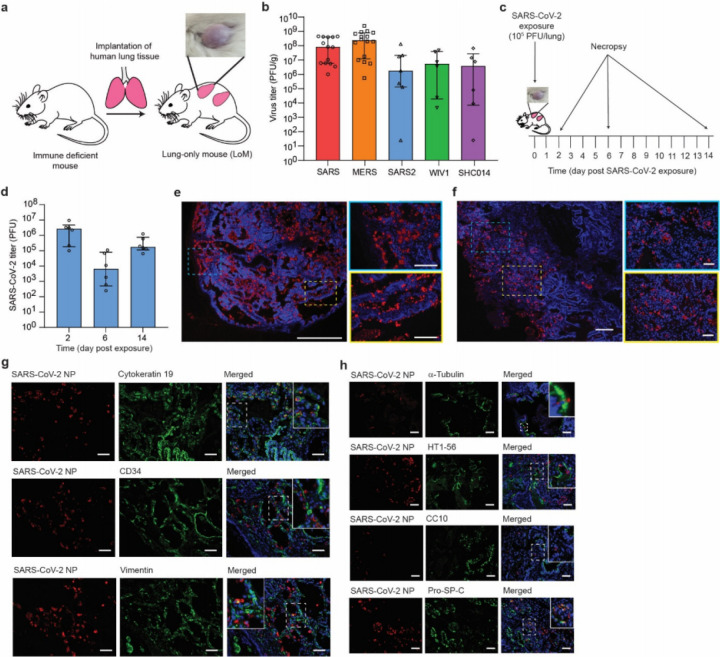
Robust replication of recently emerged human and bat coronaviruses in LoM demonstrate the potential of bat coronaviruses for direct transmission to humans and the predilection of SARS-CoV-2 for infection of human epithelial cells. a, Construction of LoM and image of a human lung implant. b, Titers of virus in the human lung tissue of LoM directly injected with SARS-CoV (n=14, red), MERS-CoV (n=16, orange), SARS-CoV-2 (n=7, blue), WIV1-CoV (n=6, green), or SHC014 (n=6, purple) as determined by plaque assay (PFU, plaque forming units). c, SARS-CoV-2 was directly injected into the human lung tissue of LoM. Human lungs were collected at days 2, 6, and 14 post-infection. d, SARS-CoV-2 titers in the human lung tissue of LoM

LoM are constructed by subcutaneous implantation of a small piece of human lung tissue into the back of immune deficient mice (Fig. 1a). Previously, we demonstrated that the human lung tissue expands to form a highly vascularized palpable implant^17^ (Fig. 1a). These lung implants contain human fibroblast, epithelial, endothelial, and mesenchymal cells that form highly relevant lung structures including cartilaginous and non-cartilaginous bronchial airways lined with ciliated and non-ciliated epithelium, alveolar sac structures, and extensive vasculature^17^ (Extended Data Fig. 1a,b). We also showed that the human lung tissue of LoMs supports replication of a diverse set of emerging and clinically relevant human pathogens including Zika virus, human cytomegalovirus, respiratory syncytial virus and MERS-CoV^17^. Recently emerged human coronaviruses have used at least two different receptors to gain entry into host cells, human angiotensin converting enzyme-2 (ACE2) and dipeptidyl peptidase 4 (DPP4). SARS CoV and SARS-CoV-2 use human ACE2 as a receptor while MERS-CoV uses DPP4^18–22^. These differences in receptor usage influence viral tropism, pathogenesis and disease progression^23^. Infection of LoM with MERS-CoV resulted in robust virus replication and infection of human epithelial, endothelial and mesenchymal cells *in vivo*^17^. These findings were consistent with the broad cellular distribution of its receptor, DPP4^24^.

Here, we first evaluated the potential of LoM to serve as a single platform to study all known recently emerged human coronaviruses and the potential of endogenous bat coronaviruses for human emergence. We first confirmed that ACE2, the receptor for SARS-CoV and SARS-CoV-2 in human cells, was present on the surface of human epithelial cells (cytokeratin 19+) in the human lung tissues of LoM (Extended Data Fig. 1c,d). Next, LoM were inoculated with recently emerged coronaviruses SARS-CoV, MERS-CoV, or SARS-CoV-2 (Extended Data Table 1). LoM supported replication of all these viruses *in vivo*. Specifically, SARS-CoV and SARS-CoV-2 infection resulted in mean virus titers of 1.76x10^8^ and 2.42x10^7^ PFU/g respectively at 2 days post-infection (Fig. 1b). Viral nucleoprotein antigen was abundantly observed in the human lung tissues of SARS-CoV and SARS-CoV-2 infected LoM (Extended Data Fig. 2a). Consistent with our previous results^17^, we also observed robust replication of MERS-CoV in the human lung tissues of LoM with mean titers of 4.79x10^8^ PFU/g in LoM human lung tissues at 2 days post-infection (Fig. 1b) and abundant viral antigen (Extended Data Fig. 2a).

Pre-pandemic bat coronaviruses WIV1-CoV and SHC014-CoV have high sequence homology to SARS-CoV, use ACE2 to infect human cells, and grow modestly in primary human airway cultures on liquid interface^4,5^. LoM were inoculated with WIV1-CoV or SHC014-CoV and virus titers in human lung tissues measured 2 days post-infection (Extended Data Table 1). WIV1-CoV and SHC014-CoV eficiently replicated in the human lung tissue of LoM with mean titers of 1.58x10^7^ and 1.48x10^7^ PFU/g, respectively (Fig. 1b) and viral antigen was readily detected in human lung tissues (Extended Data Fig. 2b). Collectively, these results demonstrate that LoM serve as single platform where all newly emerged coronaviruses SARS-CoV, MERS-CoV, and SARS-CoV-2 replicated efficiently in human lung tissue. Importantly, the fact that SARS-like bat coronaviruses WIV1-CoV and SHC014-CoV also replicated efficiently in the LoM platform indicates that bats harbor coronaviruses that are potentially capable of direct transmission to humans, thus bypassing the need for further adaptation in an intermediary host.

## SARS-CoV-2 replication in LoM

Given the state of the current COVID-19 pandemic and the urgent need to develop therapeutic and preventative approaches to control and prevent infection, we evaluated replication of SARS-CoV-2 in LoM in detail. Human lung tissues of LoM were inoculated with SARS-CoV-2 and titers of replication competent virus determined 2, 6, and 14 days post-exposure (Fig. 1c, Extended Data Table 2). High titers of replication competent virus were noted at all time points although they were highest 2 days post-infection (Fig. 1d). The distribution of virus-infected cells was determined with RNAscope (viral RNA) and immunofluorescence microscopy (viral nucleoprotein). Virus infection was widely distributed throughout the tissue with large numbers of cells positive for viral RNA (Fig. 1e) and nucleoprotein (Fig. 1f). Co-staining with a human cytokeratin 19 antibody demonstrated that SARS-CoV-2 predominantly infects human epithelial cells in the lung (Fig. 1g). Viral antigen was not detected in human CD34 expressing (endothelial) cells, and only a few human vimentin expressing (mesenchymal) cells were positive for viral nucleoprotein (Fig. 1g). To identify the epithelial cell types in the lung tissue that are susceptible to SARS-CoV-2 infection, we further identified infected cells by assessing co-localization of viral nucleoprotein with antibodies against acetylated alpha-tubulin IV (ciliated cells), CC10 (club cells), HT1-56 (alveolar type I [AT1] pneumocytes), and pro-SP-C (alveolar type II [AT2] pneumocytes) (Fig. 1h). We were able to clearly identify virus antigen in cells which expressed pro-SP-C or acetylated alpha-tubulin IV; we did not detect virus antigen in HT1-56 or CC10 positive cells (Fig. 1h). These results demonstrate that SARS-CoV-2 has limited tropism in the lung with AT2 pneumocytes and ciliated airway epithelial cells being the predominant lung cells infected by virus.

## SARS-CoV-2 pathogenesis in LoM

**Figure 2:**
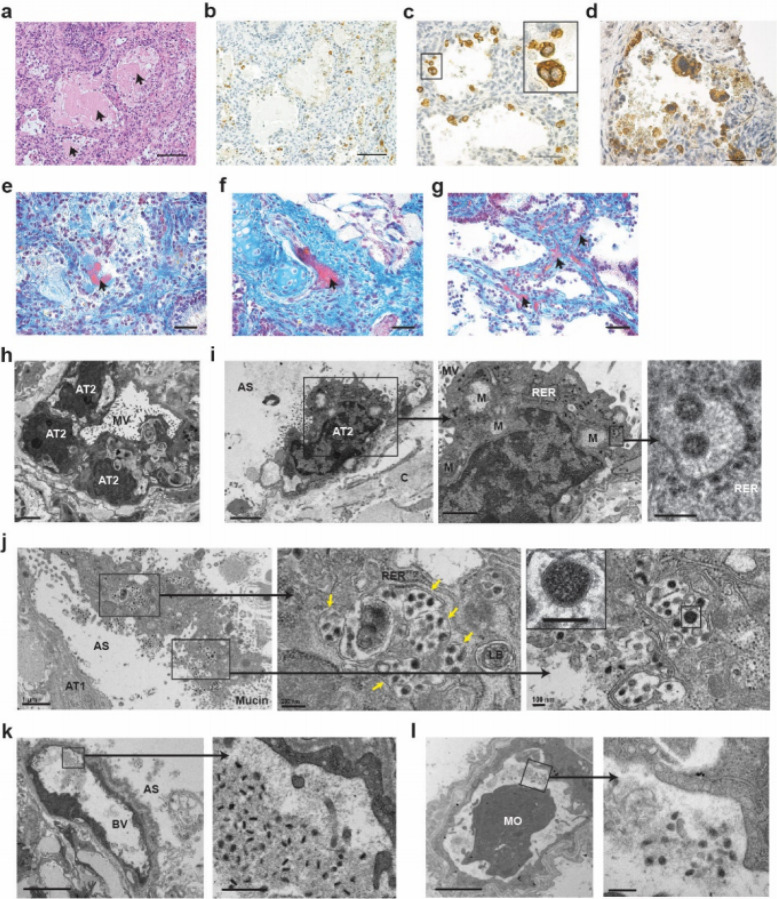
During acute infection, SARS-CoV-2 is highly cytopathic and causes extensive damage to human lung structures. a, H&E staining of a SARS-CoV-2 infected LoM human lung tissue 2 days post-exposure (scale bar, 100 um, n=6 analyzed). Globules of protein material are indicated with arrows. b-d, Immunohistochemical staining for virus nucleoprotein in LoM human lung tissue 2 days following SARS-CoV-2 exposure (positive cells, brown; b scale bars, 100 um; c and d scale bars, 50 um, n=6 analyzed). e-g, Martius Scarlet Blue staining of a SARS-CoV-2 infected LoM human lung tissue 2 days post-exposure (scale bars, 50 um; fibrin, red; collagen, blue, n=6 analyzed). Arrows indicate the presence of fibrin (red) in e, alveoli or in f and g, thrombi of occluded vessels. h-l, Electron microscopy analysis of SARS-CoV-2 infected human lung tissue two days post-exposure (n=3 analyzed). h, Uninfected AT2 cells in an alveolus-like structure. Scale bars, 2 um. i, SARS-CoV-2 infected AT2 cell. Higher magnification images of areas indicated with black boxes show virus particles with dense nucleocapsids in RER. Scale bars, 2 um (left image), 1 um (middle image), and 200 nm (right image). j, A degenerative SARS-CoV-2 infected cell in the alveolar space. Vesicles filled with virus particles in the middle image are indicated with arrows. Scale bars, 1 um (left image), 200 nm (middle image), and 100 nm (right image). k and l, Blood vessels containing virions, fibrillar protein and cell debris. In k, scale bars, 5 um (left image) and 500 nm (right image). In l, scale bars, 2 um (right image) and 200 nm (left image). AS, alveolar space; AT1, alveolar type 1 cells; AT2, alveolar type 2 cells; BV, blood vessel; C, collagen; LB, lamellar body; M, mitochondria; MO, monocyte; MV, microvilli; RER, rough endoplasmic reticulum. In c, i-l, black boxes indicate areas of higher magnification images.

To evaluate the cytopathogenic effects of SARS-CoV-2 during acute infection of human lung tissue in LoM, we used a combination of histological analysis and electron microscopy. Histopathologic analysis revealed several features of early diffuse alveolar damage that have been described in lung tissues of COVID-19 patients including the accumulation of proteinaceous exudate and fibrin in alveolar spaces, desquamation of pneumocytes, multi-nucleated cell formation, and the appearance of fibrin thrombi in small vessels (Fig. 2)^25–27^. Proteinaceous exudate, including large globules of protein material, was observed in alveolar spaces, which overlapped with areas of virus accumulation (Fig. 2a,b). As early as 2 days post-infection, desquamation of pneumocytes was also noted; there were a large number of virally infected cells fully detached or detaching from the alveolar basement membrane into the alveolar space (Fig. 2c,d). Infected multi-nucleated cells were also observed (Fig. 2c). While the formation of hyaline membranes was not noted, fibrin was detected in alveolar spaces (Fig. 2e). Importantly, we observed multiple occluded vessels containing fibrin thrombi as reported in the lungs of COVID-19 patients (Fig. 2f,g)^25–27^. Electron microscopy demonstrated the normal architecture and integrity of uninfected AT2 pneumocytes that were present in human lung tissue obtained from LoM two days post-infection (Fig. 2h). In contrast, AT2 cells containing virus particles in the same sample had swollen mitochondria with loss of matrix and cristae as well as rough endoplasmic reticula with distended cisternae, protein accumulation, and virus particles (Fig. 2i). Degenerative SARS-CoV-2 infected AT2 cells detached from the alveolar basal membrane could also be observed in the alveolar luminal space (Fig. 2j). Higher magnification revealed subcellular accumulation of virus containing vesicles indicative of virus replication and egress. Virions with electron dense nucleocapsids and distinctive crown-like spikes were observed (Fig. 2i,j). Consistent with previous reports in human airway epithelial cell cultures and port-mortem lung samples^26,28^, virions produced by human lung cells were pleomorphic in size (69 to 112 nm) and shape. Despite the extensive damage inflicted in the lung tissue by the virus, the endothelium in the majority of blood vessels was intact with tight junctions, numerous pinocytotic vesicles, and normal mitochondria and endoplasmic reticulum (Fig. 2k,l). Virions were not detected within endothelial cells in agreement with a lack of infection as per our immunofluorescence analysis (Fig. 1g and Fig. 2k,l). However, pleomorphic virions were present in capillary lumen surrounded by fibrillar protein deposits and cell debris (Fig. 2k,l). Together, these results demonstrate that acute SARS-CoV-2 infection of LoM closely resembles infection of human lung in humans and is highly cytopathic resulting in significant injury to the fragile alveolar lung structures.

**Figure 3:**
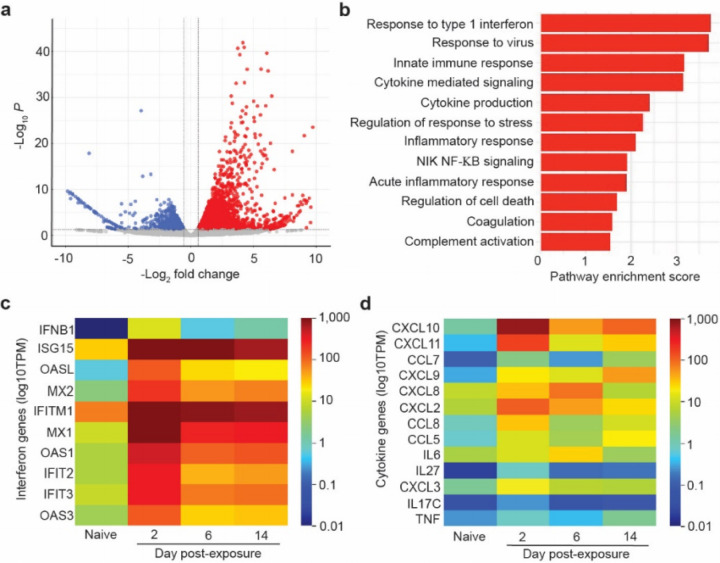
SARS-CoV-2 infection induces a strong and sustained host innate immune response in human lung tissue. a-d, RNA-sequencing analysis of human lung tissue collected from SARS-CoV-2 infected LoM. a, The plot depicts the up-regulated (red) and down-regulated (blue) genes in SARS-CoV-2 infected human lung tissue two days post-infection (n=2) compared to human lung tissue from naïve LoM (n=2). The mean - log2-transformed fold change and the multiple testing adjusted -log10 p value are shown on the x-axis and y-axis respectively. Dashed lines show the thresholds of log2-transformed fold change of 1.5 and adjusted p< 0.05. b, Gene set enrichment analysis (GSEA) identified gene sets enriched in SARS-CoV-2 infected LoM human lungs (red, p<0.05). The pathway enrichment score is shown on the x-axis. Heatmaps illustrating the expression of human c, interferon genes and d, cytokine/chemokine genes in human lung tissue collected analyzed from SARS-CoV-2 infected LoM days 2 (n=2), 6 (n=3), and 14 (n=3) post-exposure and naïve LoMs (n=4). Color scale indicates the mean log10 transcripts per million (TPM).

To determine the effect of SARS-CoV-2 infection on human gene transcription, we performed RNA-sequencing analysis of human lung tissues collected from animals 2, 6 and 14 days post-infection. Abundant viral transcripts were detected in infected lung tissue, ranging from 0.55% to 3.6% of the total reads at 2 days post-infection (Extended Data Table 3). Viral transcripts were still abundant but lower at 6 days and 14 days post-infection (Extended Data 3). Sequencing data was consistent with previously identified canonical SARS-CoV-2 transcripts^29^ and confirmed maintenance of the furin cleavage site throughout the course of infection *in vivo*. Analysis of human gene transcripts revealed 1,504 differentially expressed cellular genes between naïve and infected human lung tissue at 2 days post-exposure, the peak of infection (Fig. 3a, Supplementary Tables 1 and 2) (adjusted p value <0.05 after correcting for multiple testing). Of these, 1,043 genes were up-regulated and 461 genes were down regulated in the infected human lung tissue relative to non-infected lung tissue (Fig. 3a, Supplementary Tables 1 and 2). Notably, numerous interferon-stimulated genes (ISGs) and inflammatory cytokine genes, including pro-inflammatory cytokines genes *IL6*, *CXCL8* (IL-8), *CXCL10* (IP-10), *TNF*, and *CCL5* (RANTES) were potently induced in infected lung tissue (Supplementary Tables 1 and 2). We also observed dramatic upregulation of IFNB1 expression (>1,000 fold) at 2 days post-exposure, suggesting that this cytokine plays a key role in the antiviral response to SARS-CoV-2 (Supplementary Tables 1 and 2). Gene set enrichment analysis (GSEA) showed over 840 gene pathways significantly upregulated (p<0.05) including response to type 1 interferon (p=0.0011), response to virus (p=0.0010), innate immune response (p=0.0010), cytokine mediated signaling (p=0.0010), cytokine production (p=0.0010), response to stress (p=0.0010), inflammatory response, (p=0.0010), NIK NF-KB signaling (p=0.0011), acute inflammatory response (p=0.0035), regulation of cell death (p=0.0030), and coagulation pathways (p=0.0453) (Fig. 3b). Complement activation, which contributes to SARS-CoV pathogenesis in mouse models^14^, was also increased (p=0.0470) (Fig. 3b). Importantly, analysis of host gene expression at later time points demonstrated a sustained upregulation of antiviral and inflammatory genes that in some instances (e.g. I*SG15*, *IFITM1*, *TNF*, *CXCL9*) persisted for up to 14 days post-infection (last time analyzed) (Fig. 3c,d, Extended Data Table 4, Supplementary Tables 1 and 2). These results demonstrate that acute SARS-CoV-2 infection causes a potent and sustained upregulation of innate immune responses in virus-infected human lung tissue.

## EIDD-2801 pre-exposure prophylaxis

**Figure 4:**
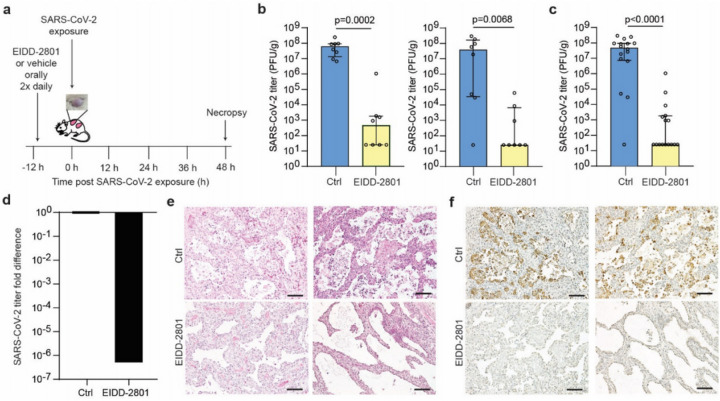
Pre-exposure prophylaxis with EIDD-2801, a broad-spectrum anti-coronavirus drug, potently prevents SARS-CoV-2 infection in vivo. a, Experimental design. LoM were administered EIDD-2801 or vehicle control 12 h prior to SARS-CoV-2 exposure and every 12 h thereafter. Virus titers in human lung tissues were measured 2 days post-exposure. b and c, SARS-CoV-2 titers in the human lung tissue of LoM administered EIDD-2801 (n=8 per experiment, yellow) or control vehicle (Ctrl, n=8 per experiment, blue) at 2 days post-exposure in two independent experiments shown b, separately and c, combined. Titers were compared with a two-tailed Mann-Whitney U test. Horizontal and vertical lines represent the median and interquartile range respectively. d, Fold difference in SARS-CoV-2 titers in the human lung tissue of LoM relative to vehicle controls. e, H&E staining and f, immunohistochemical staining for virus nucleoprotein (positive cells, brown) of human lung tissue of LoM administered EIDD-2801 (n=8 analyzed) or control vehicle (Ctrl, n=8 analyzed) at 2 days post-exposure (scale bars, 100 um).

Currently, there is no vaccine to prevent SARS-CoV-2 infection or effective therapy to treat patients with COVID-19. The ribonucleoside analog β-D-N^4^-hydroxycytidine (NHC) has been shown to broadly inhibit coronavirus infection *in vitro* in human airway epithelial cell cultures, with potent activity against SARS-CoV-2 as well as SARS-CoV, MERS-CoV, and bat SARS-like and MERS-like coronaviruses^7^. We therefore tested the ability of prophylactic EIDD-2801 (also known as MK-4482), the oral pro-drug of NHC, to inhibit SARS-CoV-2 replication *in vivo*. For this purpose, LoM were administered EIDD-2801 starting 12 h prior to SARS-CoV-2 exposure and every 12 h thereafter (Fig. 4a, Extended Data Table 5). Our results show that EIDD-2801 had a dramatic effect on virus infection, significantly reducing the number of infectious particles in the human lung tissue of EIDD-2801 treated animals in two independent experiments (Fig. 4b,c) by over 100,000 fold (Fig. 4c,d). Furthermore, in contrast to EIDD-2801 treated mice, abundant cell debris and nucleoprotein positive cells could be readily observed in the alveolar lumen of vehicle control treated mice consistent with the extensive pathogenic effects inflicted on the lung by SARS-CoV-2 (Fig.4e,f). These results demonstrate that prophylactic administration EIDD-2801 is highly effective at preventing SARS-CoV-2 infection and pathogenesis *in vivo*.

## Discussion

The zoonotic transmission of the pathogenic SARS-CoV-2 resulted in a pandemic that has inflicted significant morbidity and mortality as well as dire world-wide economic and social consequences. Herein, we describe a unique model for the *in vivo* study of human coronavirus infection particularly well suited to model distal human lung virus infection. Our results demonstrate replication of all known recently emerged human coronaviruses in LoM.

Importantly, our results demonstrate that WIV1-CoV and SHC014-CoV, two pre-pandemic endogenous bat viruses can replicate relatively efficiently in LoMs suggesting that coronaviruses circulating in bats have future pandemic potential without the need for further adaptation to the human host. We also show that acute SARS-CoV-2 infection of LoM resulted in significant lung injury and exhibited key features of the extensive pathology observed in lung tissues obtained from patients with severe SARS-CoV-2 infection, including desquamation of alveolar epithelial cells, multi-nucleated cell formation, and fibrin thrombi^25,27,30,31^. In agreement with analyses of bronchoalveolar fluid obtained from COVID-19 patients and post-mortem patient lung samples^12,32^, we also observed a significant increase in multiple ISGs in SARS-CoV-2 infected human lung tissue of LoM. In addition, we observed a robust induction in IFNB1 gene expression in human lung tissue early during acute infection followed by a decline in expression. Interestingly, an analysis of post-mortem COVID-19 patient lungs^12^ did not reveal increased IFNB1 expression and it has been shown *in vitro* that its expression is blocked during SARS-CoV infection^33,34^. These results suggest that in human lung tissue IFNB1 gene expression is induced during the acute phase of SARS-CoV-2 infection. We also observed increased expression of several human cytokine genes in LoM infected with SARS-CoV-2. A substantial number of these cytokines were also increased in the serum of COVID-19 patients and post-mortem lung tissue samples further establishing the similarities between LoM and human infection with SARS-CoV-2^12,35^. Currently, there is no vaccine to prevent or therapeutics to treat COVID-19. Pre-exposure prophylaxis approaches to infectious diseases have proven to be highly efficacious and can contribute to reduce the risk of infection. The continued global spread of the virus and its associated morbidity and mortality are strong incentives to the development of prevention strategies for COVID-19. In this regard, NHC was shown to have broad activity against human and bat coronavirus infection *in vitro*^7^. In addition, prophylactic and therapeutic administration of its oral pro-drug EIDD-2801 reduced SARS-CoV and MERS-CoV replication and pathogenesis in mice^7^. Here we show that prophylactic administration of EIDD-2801 eficiently prevents SARS-CoV-2 infection *in vivo* highlighting its potential utility as an effective prophylactic agent against SARS-CoV-2 and other past and future zoonotic coronaviruses. There are some limitations of our study including the fact that LoM do not possess the human nasal airway structures that are thought to be early sites of SARS-CoV-2 replication in humans^36^. Since LoM do not have an autologous human adaptive immune system they reflect the direct effect of viruses on their targets and bystander cells as well as their innate immune response to infection. Collectively, our results indicate that LoM reflect the pathogenic effects inflicted by SARS-CoV-2 on the human lung and demonstrate their utility as a single *in vivo* platform to evaluate and compare the replication and pathogenesis of past, present, and future pre-emergent, epidemic, and pandemic coronaviruses accelerating the development and testing of pre-exposure prophylaxis agents such as EIDD-2801.

## Methods

### Experimental design

Human lung-only mice (LoM) were used as an *in vivo* model to evaluate infection of lung tissue with recombinant coronaviruses SARS-CoV, MERS-CoV, and SARS-CoV-2 as well as full length bat coronaviruses WIV1 and SHC014^4,5,37,38^. Viruses were directly injected into the human lung tissue of LoMs and lung tissue collected either 2, 6, or 14 days post-exposure for virus titer determination and/or analysis by histology, electron microscopy, or RNA-seq.

### Construction of humanized mice

LoMs were constructed with 1–2 human lung implants by surgically implanting human lung tissue (Advanced Bioscience Resources) subcutaneously into the upper and lower back of male and female 12–21 week old NOD.Cg-Prkdc^scid^ ll2rg^tm1Wjl^/SzJ mice [NSG mice; The Jackson Laboratory] as previously described^17^. Engraftment of lung tissue was assessed by palpation and by 8 weeks post-surgery animals were ready for experimentation. Mice were housed and maintained by the Division of Comparative Medicine at the University of North Carolina-Chapel Hill and in accordance to the NIH Guide for the Care and Use of Laboratory Animals.

### Production of coronavirus stocks and infection of humanized mice

Stocks of wild-type SARS-CoV, MERS-CoV (HCoV-EMC/2012), SHC014-CoV, and WIV1-CoV were derived from infectious virus clones and were prepared and titered on Vero E6 (SARS-CoV, SHC014-CoV, and WIV1-CoV) or Vero CCL81 cells (MERS-CoV) (American Type Culture Collection) as previously described^4,5,9,17^. A clinical isolate of SARS-CoV-2 (2019-nCoV/USA-WA1/2020) was obtained from the U.S. Centers for Disease Control and Prevention (GenBank accession no. MN985325.1) and passaged twice in Vero E6 cells to create a passage 5 working stock^36^. For the infection of mice with coronavirus, the fur over the human lung tissue of anesthetized mice was shaved and virus (10^5^ PFU in 100 ul PBS or 3x10^5^ PFU in 100 ul PBS for bat coronavirus inoculations) was injected directly into the lung tissue. To evaluate the *in vivo* inhibitory activity of EIDD-2801, mice were administered 500 mg/kg EIDD-2801 or vehicle control (10% PEG and 2.5% Cremophor RH40 in water) via oral gavage starting 12 h prior to SARS-CoV-2 exposure and every 12 h thereafter. At necropsy, human lung tissues were collected, weighed, homogenized, and stored at −80°C until tittering on Vero E6 cells. Titers below the limit of the assay (50 PFU/mL) were assigned a value of 25 PFU/gram.

### H&E staining of human lung tissue

Human lung tissues collected from LoM were fixed in 10% formalin, paraffin embedded, and cut into 5 µm sections which were mounted onto Superfrost Plus slides (Fisher Scientific). Tissue sections were incubated at 60°C for 1 h, deparaffinized with xylene (2 × 3 min) and graded ethanol (100% 2 × 3 min, 95% 1 × 3 min, 80% 1x 3min, 70% 1 × 3 min), and stained with hematoxylin followed by eosin. Tissue sections were then mounted and imaged on a Nikon Eclipse Ci microscope using Nikon Elements BR software (version 4.30.01) with a Nikon Digital Sight DS-Fi2 camera. Brightness, contrast and white balance were adjusted on whole images in Adobe Photoshop (CS6).

### Immunohistochemical analysis of coronavirus infection

Immunohistochemistry was performed as previously described^17^. Briefly, fixed (10% formalin) human lung tissues collected from coronavirus-infected LoM were paraffin embedded and sectioned (5 um). Tissue sections mounted on Superfrost Plus slides (Fisher Scientific) were deparaffinized as described above. Following antigen retrieval using Diva Decloaker (BioCare Medical), non-specific binding was blocked using Background Sniper (BioCare Medical). Tissue sections were then incubated with primary antibodies against SARS-CoV or MERS-CoV nucleocapsid overnight at 4°C. Tissue sections incubated with rabbit IgG were used as isotype controls. Tissue sections were then washed in TBST and the endogenous peroxidase activity blocked with hydrogen peroxide. Tissue sections were developed using the MACH-3 polymer system (BioCare Medical) and 3,3′-diaminobenzidine (DAB) (Vector Laboratories), counterstained with hematoxylin, and mounted. Tissue sections were imaged on a Nikon Eclipse Ci microscope using Nikon Elements BR software (version 4.30.01) with a Nikon Digital Sight DS-Fi2 camera. Adobe Photoshop (CS6) was used to adjust brightness, contrast and white balance on whole images.

### Immunofluorescence analysis of SARS-CoV2 infection

Human lung tissues collected from mice were fixed in 10% formalin and paraffin embedded. Immunofluoresence staining of 5 um tissue sections was performed as previously described^17^. Briefly, following deparaffinization and antigen retrieval (Diva Decloaker), tissue sections were incubated with a 10% normal donkey serum solution with 0.1% Triton X-100 in 1x PBS to block non-specific binding. Tissue sections were then incubated overnight with primary antibodies at 4°C followed by incubation with fluorescent conjugated secondary antibodies (Supplementary Table 7). Primary antibodies were directed against SARS nucleoprotein and human cytokeratin 19, CD34, vimentin, acetylated alpha-tubulin IV, CC10, HT1-56, and pro-SP-C (Supplementary Table 7). Background autofluorescence was then quenched using a 0.1% Sudan Black B solution in 80% ethanol prior to staining with DAPI. Slides were mounted and then imaged using an Olympus BX61 upright wide-field microscope using Volocity software (version 6.3) with a Hamamatsu ORCA RC camera. Appropriate negative controls without primary antibodies were also imaged using the same exposure time as matching stained sections. Whole image contrast, brightness, and pseudocoloring were adjusted using ImageJ/Fiji (Version 2.0.0-rc-69/1.51w) and Adobe Photoshop (version CS6).

### RNA in situ hybridization (RNA-ISH) analysis of SARS-CoV2 infection

RNA-ISH was performed on 10% formalin fixed, paraffin-embedded, 5 μm sections of human lung tissues using the RNAscope 2.5 HD Reagent Kit according to the manufacturer’s instructions (Advanced Cell Diagnostics). Briefly, tissue sections were mounted on Superfrost Plus microscope slides (Fisher Scientific), heated at 60°C for 1 h, deparaffinized in xylene (2 x 5 minutes) and 100% ethanol (2x2 minutes), and air-dried. Tissue sections were then incubated with hydrogen peroxide to block endogenous peroxidases for 10 min at RT, followed by epitope retrieval (Advanced Cell Diagnostics) for 30 min in a 95°C water bath. Subsequently, tissue sections were immediately washed in double distilled water then dehydrated in 100% ethanol for 2 min before air-drying. Tissue sections were then incubated with Protease Plus (Advanced Cell Diagnostics) for 30 min at 40°C in a HybEZ hybridization oven (Advanced Cell Diagnostics). Sections were rinsed 3 times in double distilled water and then incubated with pre-warmed target probe designed to hybridize with the spike protein of SARS-CoV-2 (Cat. Number 848561, Advanced Cell Diagnostics) for 2 h at 40°C. Tissue sections were then washed and the signal amplified according to the manufacturer’s instructions and developed using alkaline phosphatase and Fast Red substrate. Tissue sections were counterstained with DAPI, mounted with Prolong® Gold (Invitrogen), and imaged on an EVOS M5000 microscope (Invitrogen).

### Electron microscopy analysis of SARS-CoV2 infection

Small pieces of human lung tissue collected from SARS-CoV-2 infected LoM at two days post-infection were fixed in 4% paraformaldehyde/2.5% glutaraldehyde in 0.15 M sodium phosphate buffer, pH 7.4, for 2 h at RT. The tissues were subsequently transferred to 10% formalin for 7 days. Specimens were washed in 0.1 M sodium cacodylate, pH 7.4, then post-fixed with 1% cacodylate-buffered osmium tetroxide for 1 h. After washing in 0.05 M sodium cacodylate buffer, pH 7.0, the samples were treated with 1% tannic acid in 0.05 M sodium cacodylate buffer for 30 min to enhance tissue contrast and preserve structure^39^. Tissue pieces were washed in deionized water, dehydrated in ethanol, and placed through two exchanges of propylene oxide before infiltration and embedment in PolyBed 812 epoxy resin (Polysciences). Semi-thin (1 µm) sections of tissue blocks were cut and stained with 1% toluidine blue in 1% sodium borate for examination by light microscopy. Ultra-thin (70 nm) sections were cut of selected regions of interest, mounted on 200 mesh copper grids and stained with 4% aqueous uranyl acetate and Reynolds’ lead citrate. Grids were observed on a JEOL JEM 1230 transmission electron microscope operating at 80kV (JEOL USA, Inc.) and images were acquired with a Gatan Orius SC1000 CCD Digital Camera and Gatan Microscopy Suite software (version 3.0, Gatan, Inc.). Virus particle sizes were measured in Fiji/Image J (version 2.0.0-rc-69/1.52p).

### Processing of human lung tissues for RNA-sequencing analysis

Human lung tissues were collected in RNAlater and kept at 4°C for 24 h prior to storage at −80°C until further processing. To isolate RNA, samples stored in RNAlater were thawed and the tissue transferred to a new tube containing 1 mm glass beads and 1 mL Trizol. Tissues were subsequently homogenized using a MagNA Lyser (Roche) for 30 sec at 6,000 rpm. In between rounds of homogenization, tissues were incubated on ice for 1 min. Following tissue homogenization, Trizol homogenate was transferred to a new tube and stored at −80°C.

### RNA-sequencing analysis

RNA was extracted from lung samples using a Trizol Plus RNA extraction kit (Thermo Fisher), quantified using a Qubit RNA assay kit and checked for quality using a Bioanalyzer RNA600 Nano kit (Agilent). RNA integrity scores were typically 7.0 and greater. 1ug of RNA was used to construct libraries for sequencing using a NEBNext Ultra II library prep kit with polyA RNA selection. Barcoded libraries were sequenced on a Novaseq 6,000 2x100 bp following manufacturer’s instructions (Illumina). Sequence quality was assessed using FASTQC (v). No issues were detected with the data and quality was typical for RNA extracted from fresh frozen material. A small amount of index hopping was detected (0.09%) due to the single indices used in the library preparation. Raw reads were mapped to the human, mouse, and SARS-CoV-2 reference genomes simultaneously (GRCh38.p13, GRCm38.p6 M25, NC_045512, respectively) using the BBsplit function in BBmap (version 38.86). This step minimized cross mapping of reads among genomes. We then mapped and quantified on a transcript and gene model basis using STAR (version 2.7.5a) and Salmon (version 1.2.1)^40,41^. Reads mapping to multiple locations were dropped from analysis. On average, in the lung tissue samples from LoM mice, 80% of the reads mapped to human (standard deviation: +/− 6%), 19% mapped to mouse (standard deviation: +/− 6%) and 1% mapped to the SARS-Cov-2 genome (standard deviation: +/− 1%). The percent of virus ranged from 0.05% to 3.4% among infected mice, with day 2 mice having the most, which is consistent with the infection titers observed. Samples from naïve mice were 95% or more human data.

### Statistical analysis

RNA-sequencing data was normalized and interrogated for changes in gene expression using DESeq2 package (version 3.1.1) in R (version 3.6.3)^42^. We focused the analysis on the naïve controls versus LoM for days 2, 6, and 14 post-infection. Wald’s tests were performed contrasting each day versus naïve controls. Because mice were sacrificed at each time point, we treated each day independently and not as a time series. *P*-values were adjusted for multiple testing using a False Discovery Rate using the Benjamini & Hochberg method^43^. Data was analyzed both jointly and within each treatment compared to naïve controls. Differential expression of outliers was assessed and found insignificant in overall effect. Graphs and summary tables were built in R using ggplot; gene set enrichment was performed using GSEA and GO analysis (tidyverse 1.3.0; PCATools 1.2.0; Sqldf 0.4–11; na.tools 0.3.1; ggbiplot 0.55; ggplot2 3.3.1; dplyr 0.8.4). Specific gene sets of interest were then interrogated for patterns of expression across treatment and time using unsupervised clustering of normalized gene expression counts. Gene Ontology (GO) analysis and visualization were performed with GOrilla^44^. Data was uploaded to the NCBI GEO archive (accession: GSE155286). Virus titers between vehicle control and EIDD-2801 treated LoM were compared with a two-tailed Mann-Whitney U test.

## Supplementary Material

1

2

## Data Availability

Gene-expression data are available at the Gene Expression Omnibus (GEO) repository (accession: GSE155286). All other data is available in the manuscript or the supplementary materials.
